# Evaluation of the Sexually Transmitted Infections Programme among Key and Priority Populations in Primary Healthcare Facilities to Inform a Targeted Response: A Protocol Paper

**DOI:** 10.3390/mps7030047

**Published:** 2024-06-05

**Authors:** Mohlago Ablonia Seloka, Edith Phalane, Refilwe Nancy Phaswana-Mafuya

**Affiliations:** 1 South African Medical Research Council/University of Johannesburg (SAMRC/UJ) Pan African Centre for Epidemics Research (PACER) Extramural Unit, Johannesburg 2092, South Africa; mohlagos@uj.ac.za (M.A.S.); edithp@uj.ac.za (E.P.); 2 Department of Environmental Health, Faculty of Health Sciences, University of Johannesburg, Johannesburg 2092, South Africa

**Keywords:** key and priority populations, sexually transmitted infections, primary healthcare facilities, STI programme evaluation, Limpopo Province, South Africa

## Abstract

Primary healthcare facilities lack routine diagnostic screening due to resource limitations and dependence on syndromic management, resulting in an unprecedented prevalence and incidence of sexually transmitted infections (STIs), particularly among key and priority populations. Specific focuses are essential to strengthen current STI control measures. Therefore, this article describes the protocol for evaluating STI programme among key and priority populations in selected primary healthcare facilities in South Africa. We will employ an exploratory, descriptive research design to assess the STI programme in terms of its facility operations, functions, scope, gaps, delivery services, STI surveillance methods, and indicators in the selected primary healthcare facilities. A purposive sample of 15–20 STI programme stakeholders will be selected from five primary healthcare facilities in Limpopo Province, South Africa. The programme evaluation will use the World Health Organization assessment checklist tool, a globally recognised and validated instrument comprising open- and closed-ended questions to assess the STI programme. This tool, known for its credibility and reliability, ensures the study’s validity. Quantitative data will be captured on STATA software (College Station, TX, USA) version 18 for descriptive analysis and presented as the mean and standard deviation for continuous variables, proportions and percentages for categorical variables. A *p* ≤ 0.05 will demonstrate a statistically significant level. Thematic content analysis will be conducted for the qualitative data using Atlas. ti software (Technical University, Berlin, Germany) version 23.1. The study’s results will inform new approaches to strengthen STI coverage, service delivery, and linkage to care.

## 1. Introduction

Sexually transmitted infections (STIs) continue to be a significant risk to public health and individuals’ reproductive and sexual health. Yet, they receive disproportionately lower resources than other epidemics [1]. Progressions in human immunodeficiency virus (HIV) prevention, treatment, and management programmes have led to an upsurge in unprotected sexual behaviour and risky practices among key and priority populations, resulting in increased STI prevalence [2,3]. This is a serious concern in a country characterised by the highest prevalence of HIV in the world [3].

Each day, around the world, over one million new STI infections occur, with roughly 4.5 million new cases of HIV, hepatitis B, and hepatitis C [3]. In the African region, the prevalence of syphilis was reported to be 13% among sex workers; meanwhile, among the general population, the prevalence was 3.2% [3]. South Africa, in the year 2017, was reported to have the highest STI rate, with an estimation of 5.8 million chlamydia cases, 7.1 million syphilis cases, and 4.5 million gonorrhoea cases [3,4]. Furthermore, it was indicated that higher cancer incidences resulted from human papillomavirus infection, with a projection of 35.6 per 100,000 cases and 15.8 per 100,000 global averages [3,4]. Consequently, research has shown that men who have sex with men (MSM), pregnant women, people living with HIV (PLHIV), and adolescent girls and young women (AGYW) aged 15–24 years old are at increased risk of acquiring STIs compared to the general population [1,5]. The latter was found to be attributed to limited access to reproductive and sexual healthcare services by key and priority populations in primary healthcare facilities, which is exacerbated by structural and social constraints [6]. Additionally, the South African National Strategic Plan for HIV, tuberculosis (TB), and STIs 2023–2028 [6] has also highlighted that the lack of routine diagnostic testing in primary healthcare facilities is the root cause of the increasing STI prevalence and its negative impact on sexual and reproductive health.

A synergistic relationship between STIs and HIV has been demonstrated in a few studies [7,8]. For example, STIs were reported to contribute to the shedding of genital portions through non-ulcerative and ulcer-causing STIs, increasing the transmission and acquisition of HIV [9,10]. Moreover, both infections can result in psychological factors such as feelings of shame, stigma, and diminished self-esteem. It has been reported that these psychological factors are a result of gender-based violence and relationship instability and have the potential to hinder the efficiency of the partner notification process [6].

On the other hand, South Africa was indicated to have only three sentinel sites, which are in the Western Cape, KwaZulu Natal, and Gauteng Provinces [11,12]. These sites are responsible for managing STI cases [11,12]. Within these facilities, pregnant women receiving antenatal care, regardless of their HIV status, are prioritised over the general population as well as key and priority populations [13,14]. Clinics dedicated to men’s health in South Africa are also limited, irrespective of the high prevalence of male urethritis syndrome [14]. Moreover, most primary healthcare facilities in South Africa and other resource-limited countries use a syndromic case management method as a standard treatment regimen for STI syndromes [6,15]. This method is based on healthcare providers’ recognition of patients’ observable clinical signs and symptoms rather than using laboratory diagnostics [15]. Nyemba et al. [16] and Johnson et al. [17] have indicated that this approach may only treat symptomatic STIs, and this could lead to an increased risk of drug resistance resulting from over-treatment of undiagnosed asymptomatic STIs. Hence, improved STI diagnostic measures that can screen both asymptomatic and symptomatic STIs are needed to prevent unprecedented STIs and adverse health effects.

Alternative diagnostic and treatment methods, such as partner notification services and point-of-care testing, mainly using Gene Xpert and multiplex real-time polymerase chain reaction tests, to name but a few, are essential to address the limitations of syndromic case management. However, limited resources, infrastructure, and financial support hinder their success. To advocate eliminating STIs as a public health challenge by 2030 [3], improving STI diagnostic testing skills in resource-limited countries is of utmost importance. There is also a significant need to enlarge primary prevention efforts, advance STI screening accessibility, and eliminate social and structural hindrances among key and priority populations [3]. In support of the above, the proposed protocol describes how the STI programmes among key and priority populations in selected primary healthcare facilities in Limpopo Province, South Africa will be conducted. 

## 2. Methods

### 2.1. Study Design

An exploratory descriptive research design to evaluate the STI programme in terms of its facility operations, functions, scope, gaps, and delivery services in the selected primary healthcare facilities will be conducted. The chosen methodology will enhance the understanding of stakeholders’ views on STI control services and outcomes. It will also describe the existing STI programmes, initiatives, surveillance systems, structures, and policies in priority areas, needs, gaps, and improvement opportunities. Overall, the proposed research will offer insights and contextually relevant findings, benefiting research, policy, and practice. In addition, the research design will create an opportunity to explore and obtain more detailed evidence on how STI services are provided to key and priority populations in rural primary healthcare facilities. Therefore, we aim to generate new ideas and expand knowledge in that context.

### 2.2. Study Setting

The proposed study will be conducted in the Limpopo Province of South Africa, Capricorn district, in the Mankweng rural area. Limpopo Province comprises five districts: Waterberg, Sekhukhune, Vhembe, Mopani, and Capricorn. Within the Capricorn district, Polokwane City is the most populated local municipality, where Mankweng rural area is located [18]. Mankweng is located 30 km east of Polokwane Central Business District in Limpopo Province. There are 38 villages in the region, with an estimated 80248 people within the Mankweng Traditional Council [19]. The Mankweng area has an approximately 87.8:100 male-to-female ratio [19]. According to the literature, STIs are not yet one of the Limpopo Province Strategic Plan Priorities [20]. The Capricorn district was indicated to be the lowest among the other districts in the province concerning cervical cancer screening, at 28%, and antenatal first visit booking [21,22].

The study will focus on five primary healthcare facilities with diverse views from stakeholders working at two provincial tertiary hospitals and three clinics. The programme evaluation or inventory of services on the control of STIs will be conducted using the facilities mentioned earlier. We chose these primary healthcare facilities because they are the most under-researched regarding STI control services among key and priority populations. Key and priority populations face unequal challenges in accessing healthcare services. Thus, they are at increased risk of acquiring and transmitting STI and HIV infections. In addition, the difference between these primary healthcare facilities is noted in terms of operational procedures and provision of services; they cannot be presumed to be homogenous.

## 3. Procedure

The programme evaluation and inventory of services will be conducted in line with the WHO assessment tool, 2015 [23]. The WHO assessment tool was established to support and assist countries in prioritising their critical surveillance activities by utilising developed and standardised reporting forms/checklists [23]. Moreover, this WHO assessment tool will facilitate planning, monitor trends, strengthen existing systems, and identify how to optimise service delivery and strengthen STI programmes [23]. The permission to reproduce or translate the WHO STIs Assessment Tool 2015 was granted (request ID: 202402270).

### 3.1. Sampling

Purposive sampling will be utilised to select five primary healthcare facilities. Within each primary healthcare facility, we will recruit four STI stakeholders (STI programme coordinators/managers, clinic supervisors, internal programme staff, beneficiaries, and the nursing sisters) per facility. A minimum of 15–20 STI stakeholders from the five primary healthcare facilities will be interviewed. According to the researchers’ previous experience and the literature, it is estimated that a sample of 15–20 stakeholders would provide rich and context-specific information [24,25]. Smaller sample sizes can extract in-depth, valuable information from the individuals participating in programme evaluation [26]. It will enable an exploration of possible similarities and differences in views regarding STI outcomes within the primary healthcare facilities involved. The sample size may change depending on when saturation is reached. Snowball sampling could be employed if it is necessary to enlarge the sample size if the intended goal has not yet been achieved.

#### Inclusion and Exclusion Criteria

Table 1 below provides more detailed information on the inclusion and exclusion criteria for stakeholders participating in the STI programme evaluation.

### 3.2. Data Collection

The STI programme evaluation and inventory of services will be carried out using the WHO 2015 Assessment Tool [23]. Table 2 and Table 3 outline the step-by-step procedures for evaluating the STI programme, starting with pre-assessment preparation, which includes reviewing the surveillance methods, STI indicators, and how data are used. The STI indicators will consist of the number/percentage of STI screening/testing, STI diagnosis, linkage to care, STI treatment, partner tracing, STI incidence/prevalence, condom distribution/utilisation, and uptake. This will facilitate understanding of the possible advantages and risks of the STI programme evaluation. Interested stakeholders will receive detailed information about the study’s objectives and procedures to make an informed decision before participating. Along with this, consent forms and information letters will also be distributed. Consequently, each interested stakeholder will voluntarily give a written informed consent before participating in the study. Programme evaluation will utilise open and closed-ended questions at a time and location convenient for participants to gain insights about the facility’s STI programme resources, personnel, STI programme activities and components, the nature of the programme data collected, barriers, and facilitators. Engagement with each participant will range from 15 to 30 min. Furthermore, the researcher will undergo training in both qualitative and quantitative methods to effectively engage with different stakeholders during the process.

### 3.3. Statistical Analysis

Quantitative data will be analysed using STATA software (College Station, TX, USA) version 18. The chi-square test will be used to analyse categorical variables and report proportions (n) and frequencies (%). Continuous variables will be analysed using an independent *t*-test and presented as mean and standard deviations. The statistical significance level will be set at *p* ≤ 0.05. Qualitative data will be captured and analysed using Atlas ti software (Technical University, Berlin, Germany) version 23.1. Thematic content analysis will be used to analyse open-ended questions.

### 3.4. Ethical Considerations

The proposed protocol paper forms part of the doctoral research project of the author, which has received approval from the Research Ethics Committee of the University of Johannesburg (REC-2589-2024). Furthermore, this study is a component of a larger project within the South African Medical Research Council (SAMRC)/UJ Pan African Centre for Epidemic Research (PACER) Extramural Unit funded project, namely “Harnessing Big Heterogeneous Data to Evaluate the Potential Impact of HIV Responses Among Key Populations in Generalized Epidemic Settings in Sub-Saharan Africa” (REC-1504-2022).

## 4. Expected Results

The study will describe the STI programme evaluation process among key and priority populations in selected primary healthcare facilities in Limpopo Province, South Africa. This will be addressed through an STI programme evaluation to discover the availability, accessibility, affordability, and quality of STI services. Moreover, STI outcomes, such as the number/percentage of STI screening/testing, STI diagnosis, linkage to care, STI treatment, partner tracing, STI incidence/prevalence, and condom distribution/utilisation will be assessed. This evaluation will provide evidence on whether STI programmes are accomplishing positive outcomes. This will identify opportunities to support staff and managers in achieving quality development and ideal utilisation of resources to improve the programme and make modifications as/where needed. The evaluation outcome will strengthen the STI programme’s future development and effectiveness. The outcome of the evaluation will also play a role in contributing to evidence-based data for governance and policy making. Moreover, it will significantly address sustainable development goals such as reducing inequalities and promoting good health and well-being, especially among key and priority populations.

## Figures and Tables

**Table 1:** Inclusion and exclusion criteria of the stakeholders/participants for STI programme evaluation participation.

Participants	Inclusion Criteria	Exclusion Criteria
STI programme coordinators/managers, clinic supervisors, internal programme staff, beneficiaries, and nursing sisters	➢ 18 years and older. ➢ At least one year of work experience providing STI services or the utilisation of STI services in the last year. ➢ Consent to participate and be audio recorded in the study.	➢ Below 18 years of age. ➢ Less than one year of work experience in providing or utilising STI services.

STIs-sexually transmitted infections.

**Table 2:** A WHO pre-assessment preparation checklist tool for sexually transmitted infections surveillance system/programme.

Question/s		Yes/No/Don’t Know	Comments	Potential Action
Pre-assessment preparation	(A) “Is there a need to assess the surveillance system”? (B) “Is there political support to assess the surveillance system”? (C) “Is there technical capacity to conduct the STI surveillance assessment and analyse the results”? (D) “Are STI surveillance assessment outcomes likely to be acted upon”? (E) “Are there potential partners and collaborators who would be useful in conducting STI surveillance assessment”?			
Assessing the STI surveillance system	(A) “Describe the current system (Identify gaps, challenges, and weaknesses)”. (B) “Propose potential actions to address gaps and weaknesses”			
STI indicators	(A) “Which components of STI surveillance are currently implemented”? (B) “Which STI syndromes are reported”? (C) “Which STI etiologies are reported”? (D) “Are STI data reported reliably”? (E) “How much disaggregation is reported”?			
Surveillance methods	(A) “For which populations is syphilis screening offered routinely”? (B) “What methods are used to obtain STI data”? (C) “How are new cases reported”? (D) “Who in the health sector is expected to report”?			
Maximising the impact of surveillance	(A) “Is reporting monitored for completeness, timeliness, quality, and confidentiality”? (B) “How are STI data analysed”? (C) “Are STI data used to strengthen prevention and control programmes”?			

Adapted from (WHO, 2015), STIs-sexually transmitted infections.

**Table 3:** A WHO post-assessment preparation checklist tool for STI programme improvement.

Question/s	Comments
“Which components of STI surveillance are currently implemented”? (1) Case Reporting; (2) Prevalence assessments; (3) Etiology studies; (4) Antimicrobial resistance monitoring; (5) Is Pap smear conducted routinely	
“Which STI syndromes are reported”? (1) Genital ulcer disease; (2) Urethral discharge; (3) Vaginal discharge; (4) Lower abdominal pain; (5) Anorectal discharge; (6) Other (specify)	
“Which STI etiologies are reported”? (1) Syphilis case reports (new cases); (2) Syphilis prevalence (from routine screening); (3) Gonorrhoea (new cases); (4) Congenital syphilis (new cases); (5) Other STIs (specify)	

Adapted from (WHO, 2015), STIs-sexually transmitted infections.

## Data Availability

No data can be shared now as this is still a protocol paper. As the research develops and later stages of data collection are completed, information and instructional resources will be made available through the corresponding author, R.N.P.-M., upon request.
